# Ultrafast Infrared-to-Visible Photon Upconversion on Plasmon/TiO_2_ Solid Films

**DOI:** 10.1021/acs.jpclett.3c01208

**Published:** 2023-06-30

**Authors:** Xianshao Zou, Robert Bericat Vadell, Bin Cai, Xinjian Geng, Ananta Dey, Yawen Liu, Axel Gudmundsson, Jie Meng, Jacinto Sá

**Affiliations:** ‡ Qingdao Innovation and Development Base, Harbin Engineering University, Qingdao, Shandong 266000, People’s Republic of China; § Physical Chemistry Division, Department of Chemistry, Ångström Laboratory, Uppsala University, Box 523, 751 20 Uppsala, Sweden; ∥ Division of Chemical Physics, Lund University, 221 00 Lund, Sweden; ⊥ Peafowl Plasmonics AB, Uppsala 756 51, Sweden; # Institute of Physical Chemistry, Polish Academy of Sciences, Marcina Kasprzaka 44/52, 01-224 Warsaw, Poland

## Abstract

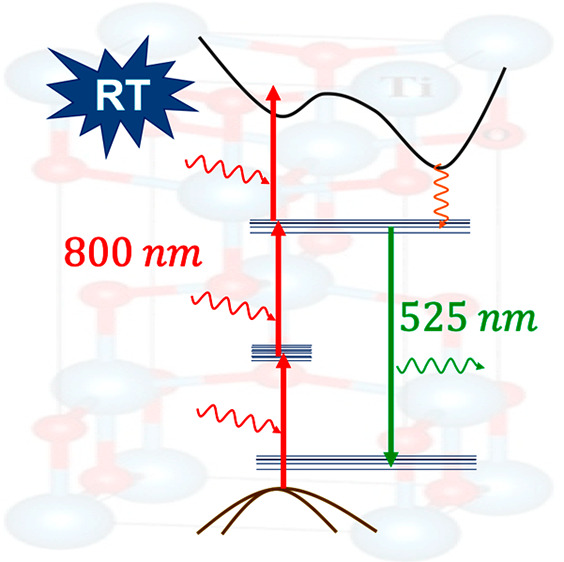

Optical upconversion via a multiphoton absorption process converts incoherent low-energy photons to shorter wavelengths. In this contribution, we report a solid-state thin film for infrared-to-visible upconversion composed of plasmonic/TiO_2_ interfaces. When excited at λ = 800 nm, three photons are absorbed, leading to the excitation of TiO_2_ trap states into an emissive state in the visible domain. The plasmonic nanoparticle enhances the light absorption capabilities of the semiconductor, increasing emission efficiency by 20 times. We demonstrate that the plasmonic nanoparticle only changes the optical absorption of the semiconductor; i.e., the process is purely photonic. The process occurs in the ultrafast domain (<10 ps), contrasting with molecular triplet–triplet exciton annihilation, the commonly used method in photon upconversion, in the nano- to microsecond time scales. The process utilizes pre-existing trap states within the semiconductor bandgap and involves three-photon absorption.

According to the United Nations (UN) 2021 global status report for buildings and construction, in 2020, the energy consumption for the construction and operation of buildings was 149 EJ or 36% of the worldwide demand and was responsible for 11.7 gigatons of carbon dioxide (more than 35% of global energy-related emissions).^1^ The values are, however, lower than what was forecasted, reflecting the impact of pandemic-related lockdowns and the precarious ability of many households and businesses to maintain and afford energy access. Buildings are significant contributors to the acceleration of climate-related issues. Any action to decrease their energy consumption would significantly impact efforts to meet the 2015 Paris Agreement commitments.

The glass of buildings allows heat to escape more readily than most other building materials. In fact, given the same window and wall area, the window will allow up to 8 times more heat escape.^2^ This is also true for heat entering the building through solar infrared radiation. The issue has led to an increased discussion on introducing legislation banning glass and steel in skyscrapers, most famously by the former New York mayor Bill de Blasio.^3^ There are two dominant solutions to prevent infrared from entering the building, namely, passive and dynamic glazing. Passive glazing uses coatings that reflect infrared radiation, for example, an ultrathin silver layer. Higher rejection of infrared increases cost and decreases glazing light transmission.^4^ The dynamic glazing, from which the electrochromic version stands out as the most promising application for buildings, reflects infrared light by electrically charging a semiconductor layer. The system is dynamic, meaning that it can be switched back and forward depending upon the level of infrared available. The system provides up to 20% energy savings at the highest infrared rejection level to cool the building.^5^ However, the highest level suppresses significant amounts of visible light from entering the building, potentially affecting work and life quality inside^5^ and prohibiting its use in historic and protected buildings. Additionally, the system is still relatively expensive and needs electric cabling, limiting its applicability to new buildings.

Optical upconversion is a process that converts two or more low-energy photons into a single high-energy photon. It has many applications, including biological imaging, night vision, multidimensional displays, and photovoltaics.^6^ It can also be used in glazing to convert nefarious infrared radiation into desirable visible light, removing undesirable heating from entering buildings without affecting visible light transmission. The most common method is triplet–triplet annihilation, where long-lived atomic or molecular excited states store the photon energy, which can then reach a higher energy state through energy transfer or subsequent absorption, leading to the emission of a high-energy photon.^7−9^ The upconversion process via triplet–triplet annihilation remains a hot and active research topic.^10^ The most efficient systems operate in liquid, permitting fast diffusion of a sensitizer and an annihilator and requiring inert conditions to function. In particular, the exclusion of oxygen is essential because molecular oxygen is an efficient triplet state quencher. Hence, triplet–triplet annihilation upconversion process systems require substantial optimization to transfer successful concepts functioning well in solution to materials. Some systems have been realized in a solid state/gel environment and fully polymer-integrated systems,^11−14^ but the quantum yield remains low compared to the liquid systems. Moreover, few polymers or gels have suitable compatibility, stability, and optical inertness.

As mentioned, molecular systems are an elegant solution. Still, they need to be made into materials, increase their stability, and reduce their fast triplet deactivation through vibrational relaxation operating at similar times as photon upconversion..^15^ Upconversion luminescence from lanthanide emitters through energy migration has been proposed.^16−18^ The most effective systems combine lanthanide nanoparticles with molecules, enabling control of lanthanide triplet exciton dynamics. This is an exciting technological platform for developing devices because the systems are materials and offer higher chemical stability. However, energy-migration-mediated upconversion requires stringent experimental conditions, such as high-power excitation and unique migratory ions in the host lattice.^17^ These issues must be improved to enable its transfer to commercial devices.

It is clear that novel photon upconversion mechanisms are needed, of which one mechanism was proposed recently by Lu and co-workers.^19^ They reported upconversion plasmonic lasing from an organolead trihalide perovskite nanocrystal with low thresholds. The system converts near-infrared photons into green photons, taking advantage of the perovskite intraband state and the optical absorption enhancement from plasmonic excitation.

Herein, we build on a similar concept by taking advantage of TiO_2_ intrabandgap energy states to perform photon upconversion involving three-photon absorption. The effectiveness of the process can be significantly increased by the presence of plasmonic nanoparticles, which enhance TiO_2_ light uptake without affecting the photophysical process.

## Methods

*Sample Preparation*. The Au nanoparticles were synthesized by the method published by Turkevich et al.^20^ A commercial titania paste (Ti-Nanoxide T/SP, Solaronix) was diluted in ethanol (100 mg/mL) and then spin-coated on a pre-cleaned thin glass substrate with a speed of 2000 rpm for 30 s. The film was then transported to the oven at 773 K for 30 min. After annealing, 10 mL of Au nanoparticle solution was sprayed on a 15 × 15 mm TiO_2_ film at 373 K. After that, the samples were put into the oven at 773 K for 30 min.

*Sample Characterization*. The ultraviolet–visible (UV–vis) absorption was determined by an Agilent 8453 UV–vis spectrophotometer. Steady-state photoluminescence (PL) was conducted on an Edinburgh FS5 spectrofluorometer. The dynamic light scattering (DLS) Malvern NanoS system measured the gold nanoparticle size. The X-ray diffraction (XRD) measurements were collected on a Siemens D5000 θ–2θ with Cu Kα (λ = 1.54060 Å) at 45 kV and 40 mA. A measurement range of 15–70° with a step size of 0.05° and a scan speed of 2 s per step was applied in this measurement. Scanning electron microscopy (SEM) was used to establish TiO_2_ morphology and validate the average size of Au nanoparticles. The measurements were performed on cover glasses and measured in LEO 1550 (Zeiss) SEM.

*Time-Resolved Characterization*. The transient energy-resolved photoluminescence measurements were carried out on a streak-camera system. A femtosecond fiber laser system generated the excitation laser beam with a wavelength of 1030 nm (Jasper 10, Fluence Sp. z o.o.). After passing an optical parametric amplifier (Harmony, Fluence Sp. z o.o., Poland), the laser beam can be tuned to 320, 400, and 800 nm with a pulse duration of 300 fs and a repetition rate of 200 kHz. Then, the laser beam was focused on the sample at an angle of 45° by one convex mirror with a focusing length of 100 mm. The PL emission was collected and collimated by a lens with a focusing length of 50 nm. Afterward, another lens focused the beam to a monochromator with an input slit with a width of 100 μm and a grating of 50 lines/mm (Chromex). The PL at a selected wavelength range was then sent to the streak camera (C5680 + M5675, Hamamatsu). The streak camera was synchronized by a delay unit (C6878, Hamamatsu) connected to the optical fiber laser system. Ultimately, the signal was captured with a digital camera (C4742-95, Hamamatsu). It is worth noting that the background signal and camera sensitivity were corrected after measuring in the data processing.

Transient infrared absorption spectroscopy (TIRAS) detected charge transfer from the plasmon to TiO_2_. A 3 kHz repetition rate pulsed laser with a pulse duration of 40 fs was generated by a Libra Ultrafast Amplifier System (Coherent). The output beam at 795 nm was split and sent to two optical parametric oscillators (OPO and TOPAS-PRIME from Light Conversion). The OPOs generate the pump beam (550 nm) and the probe light in the mid-infrared (mid-IR, 4200–4800 nm). The pump and probe pulsed beams are then sent to the TIRAS setup (Helios, Ultrafast Systems). A Horiba iHR 320 spectrometer is used for measurements in the mid-IR detector. Transient absorption spectroscopy (TAS) detected changes in plasmonic material and TiO_2_ electronic structure. We used the same TIRAS laser system and created an UV–vis probe light with a CaF_2_ crystal. The pump propagated through the delay stage and then crossed a chopper, where the repetition rate of it is lowered from 3 to 1.5 kHz.

**Figure 1:**
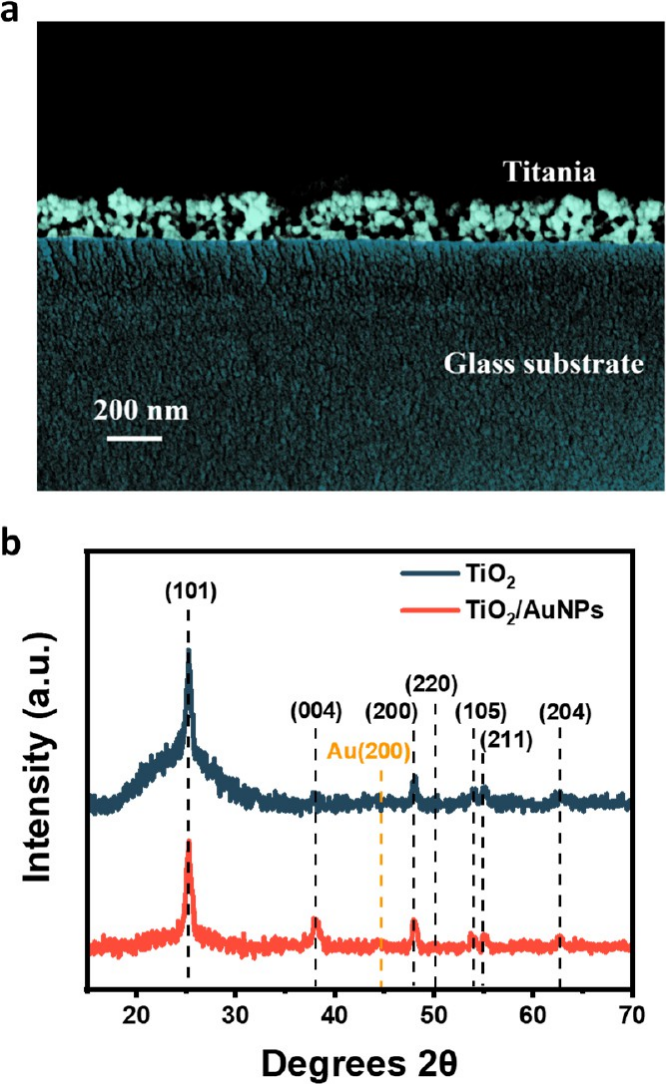
Morphologic characterization of the films: (a) SEM of the TiO_2_ film cross section and (b) XRD of TiO_2_ and Au/TiO_2_ films.

**Figure 2:**
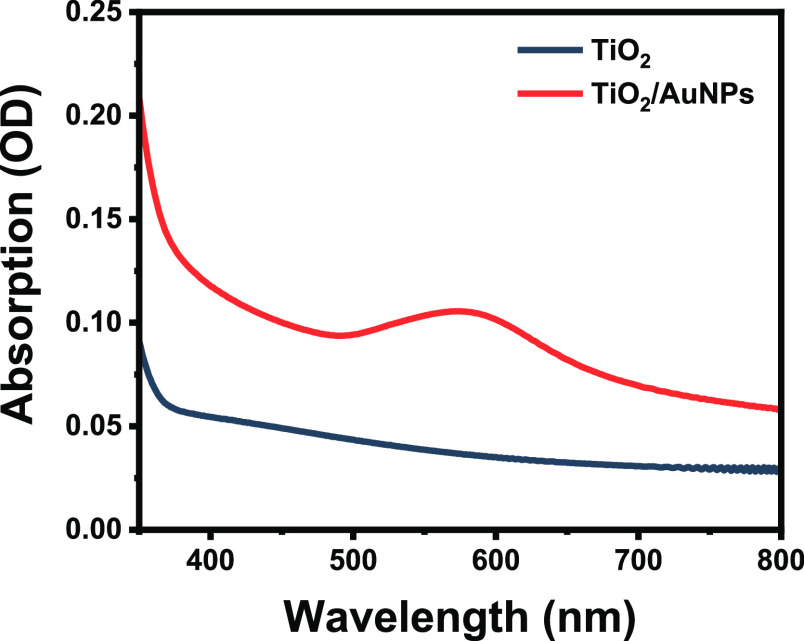
Optical absorption of TiO_2_ and Au/TiO_2_ films.

The cross-sectional analysis of the TiO_2_ films prepared via spin coating using commercially available Solaronix paste and, subsequently, annealed at 773 K for 1 h was determined by SEM shown in Figure 1a. The SEM micrograph shows granularity as a result of TiO_2_ particles and a ca. 200 nm film thickness. After annealing, the films become highly transparent in the visible region (Figure 2). The XRD revealed the characteristic peaks for anatase and rutile, with anatase with an abundance of 80% (Figure 1b), as expected from formulations with P25 powders.^21^

The Au nanoparticles with 8–10 nm determined by DLS (Figure S1a of the Supporting Information) and confirmed by SEM (Figure S1b of the Supporting Information) were deposited via spray deposition followed by annealing at 773 K for 1 h. SEM and XRD of the Au-modified films could not establish the presence of the nanoparticles, and thus, UV–vis absorption was carried out. Figure 2 shows the characteristic peak for Au localized surface plasmon resonance (LSPR) centered at 550 nm. The spectrum also shows the absorption of TiO_2_ trap states throughout the visible region.

**Figure 3:**
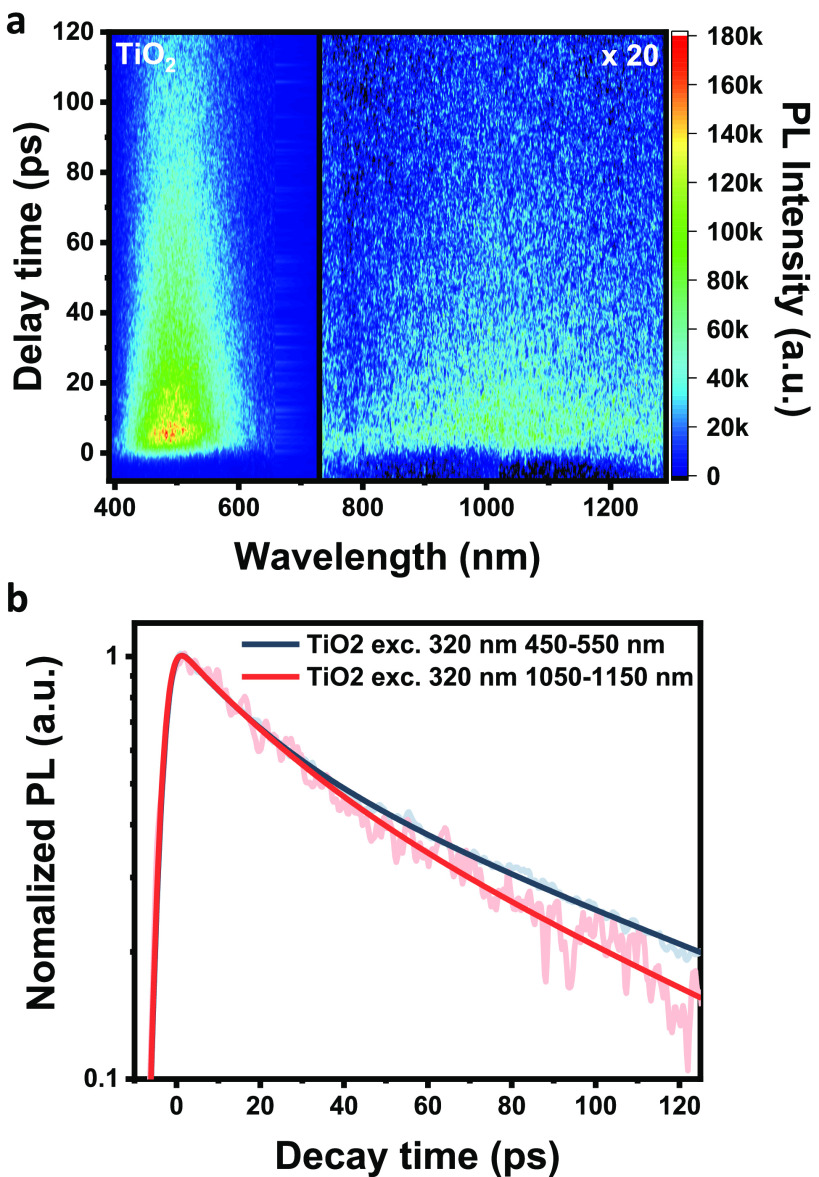
Transient energy-resolved photoluminescence of TiO_2_ was excited at 320 nm. (a) Contour map is dominated by the emission centered at 525 nm. Still, a broad detectable signal between 800 and 1200 nm related to trapped hole recombination confirms the existence of the state. (b) Kinetic traces of TiO_2_ photoluminescence excited at 320 nm: (black trace) extracted at the primary emission signal (450–550 nm) and (red trace) extracted in the infrared region (1050–1150 nm).

Room-temperature excitation of TiO_2_ at 320 nm (excitation of O^2–^ → Ti^4+^)^22^ revealed two emissive states (Figure 3a), with the most prominent state centered at 525 nm, ascribed to trap electron and trap hole charge recombination,^23^ and a weaker state in the infrared region (800–1200 nm).^22,24,25^ A detailed photophysical study of TiO_2_ and Au/TiO_2_ after TiO_2_ bandgap excitation was performed in our previous publication,^26^ and its therefore outside this contribution. However, the kinetic trace analysis presented in Figure 3b is consistent with what has been measured before but, as it will be shown, very different from what happens when exciting below the bandgap energy.

The difference between the absorptions before and after pumping gives the transient absorption. Although several papers have claimed that the positive signal comes from the absorption of the excited state, similar decays in positive and negative regions are shown in Figure S3 of the Supporting Information, indicating that the positive signal would not be the excited state absorption (ESA). The absorption difference has been published by Zhang et al.^27^ After pumping, the absorption will become broad, which leads to a positive signal in transient absorption.

**Figure 4:**
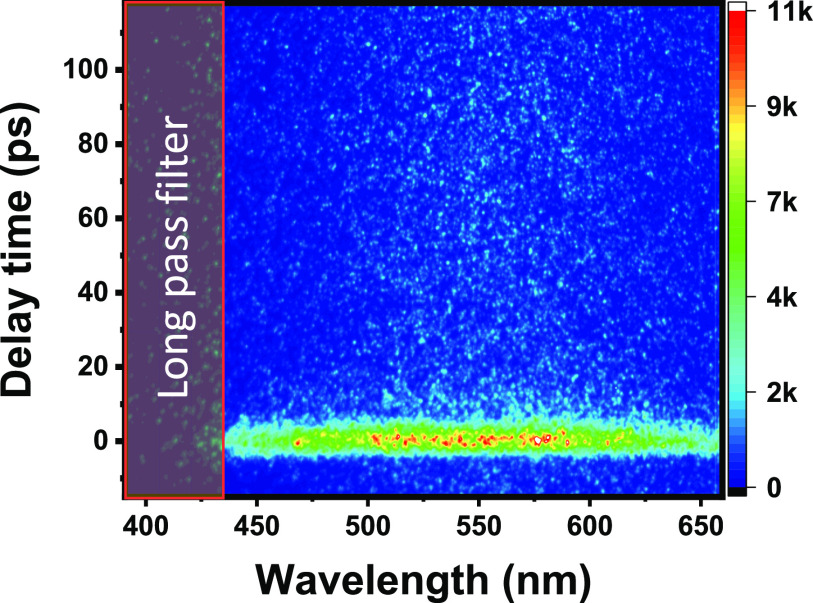
Time- and energy-resolved photoluminescence of TiO_2_ excited at 400 nm.

**Figure 5:**
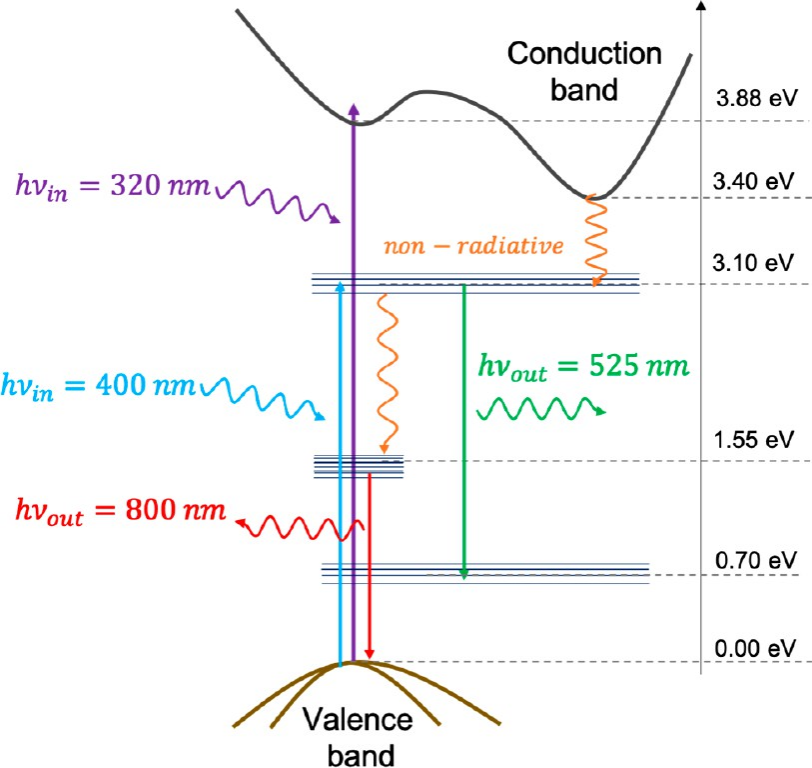
TiO_2_ energy level diagram describing the mechanism responsible for green and infrared emission.

To determine the energy levels involved in the 525 nm emissive state, the film was excited at 400 nm (sub-bandgap energy). Figure 4 shows that the emissive state is detectable even when sub-bandgap energies are used, confirming the existence of populatable trap states below the TiO_2_ conduction band. Figure 5 shows the energy level diagrams of TiO_2_ (indirect bandgap semiconductor) states responsible for the 525 and 800 nm states. Briefly, there is a trap state located ca. 0.3 eV below the edge of the conduction band of TiO_2_ that can be filled with electrons either directly or from the decay of conduction band electrons that were promoted after bandgap excitation. From there, electrons recombine preferentially with shallow trapped holes, leading to the 525 nm emission, or trap holes, resulting in 800 nm emission. The addition of gold nanoparticles increased the photoluminescence quantum yield when excited at 320 and 400 nm as a result of increased photon absorption by TiO_2_ as reported elsewhere.^23^ Adding a dielectric like ZrO_2_ had an insignificant effect on the photoluminescence process quantum yield.

**Figure 6:**
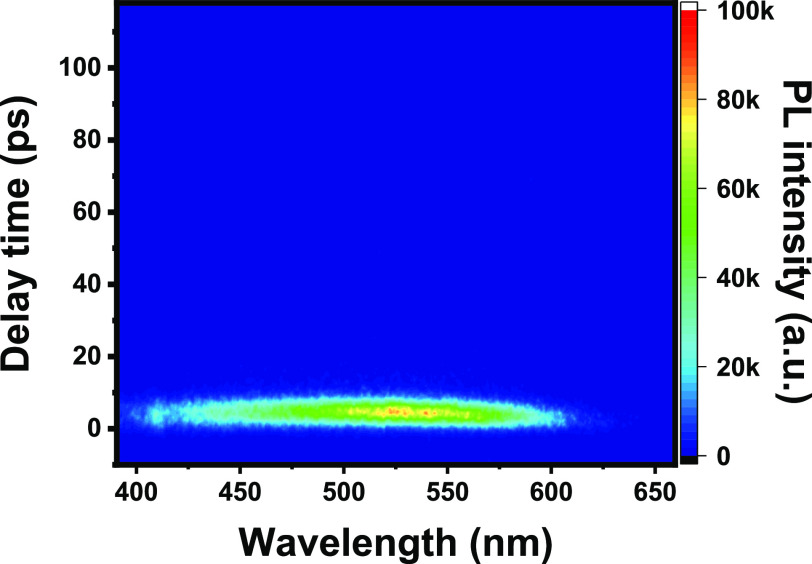
Transient photoluminescence signal of TiO_2_ after 800 nm excitation at room temperature, recorded with a streak-camera setup.

Figure 6 shows the TiO_2_ photoluminescence signal upon excitation at 800 nm at room temperature. The time- and energy-resolved photoluminescence map shows a clear emission signal between 400 and 600 nm with a maximum of 525 nm, resembling the signal observed when TiO_2_ was excited at 320 and 400 nm.

Analysis of the time component of the emission centered at 550 nm revealed an ultrafast radiative relaxation that ceases within 10 ps,^25^ independent of the excitation wavelength being at 400 or 800 nm. The streak-camera instrument response of about 5–7 ps precludes deeper analysis of the temporal response of the signal. However, it suggests a mechanism in the ultrafast domain compatible with multiphoton involvement mediated by real rather than virtual trap states.

**Figure 7:**
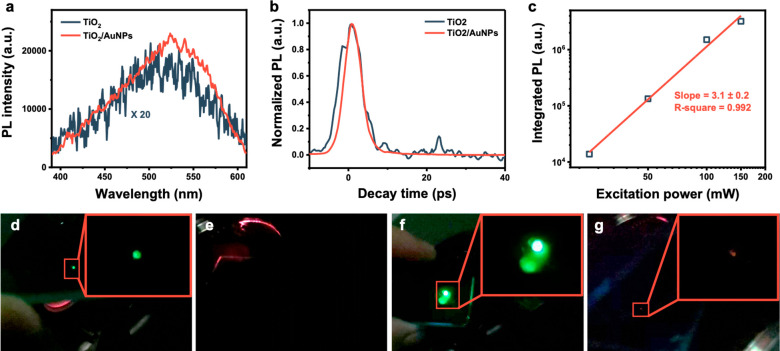
Transient photoluminescence studies: (a) comparison of the cumulative transient photoluminescence signal between TiO_2_ and Au/TiO_2_ after 800 nm excitation at room temperature, recorded with a streak-camera setup, (b) kinetic traces of the TiO_2_ and Au/TiO_2_ emissions after 800 nm excitation extracted at 525 nm, (c) behavior of the integrated photoluminescence intensity signal as a function of laser power intensity, and (d–g) photographs revealing the emission signal emanating from the samples at different temperatures excited at 800 nm: (d) TiO_2_ emission at 77 K, (e) TiO_2_ emission at 295 K, (f) Au/TiO_2_ emission at 77 K, and (g) Au/TiO_2_ emission at 295 K. Note that the color of the emission detected from Au/TiO_2_ at 297 K is green, but as a result of the weakness signal, a digital camera could not adequately image it.

A similar signal was obtained when Au was added to the TiO_2_ surface, still with a photoluminescence intensity 20 times more intense (Figure 7a). The strength of the photoluminescence signals becomes more pronounced at low temperatures (panels d–g of Figure 7). Analysis of the kinetic traces extracted at 525 nm revealed an ultrafast decay of <10 ps unaffected by the presence of Au nanoparticles (Figure 7b). The shape of the emission is sharp, resembling laser light emission signals after population inversion.

The 800 nm excitation does not overlap with the Au LSPR. Nevertheless, the wavelength can excite plasmon resonance, as confirmed by ultrafast TAS experiments. The TAS of Au/TiO_2_ was excited at 800 nm (Figure S2 of the Supporting Information) and had the characteristic bleach at the LSPR maximum and positive absorption, commonlly called a “winglet”, to the blue of the LSPR absorption.^28,29^ Note that the winglet to the red of the bleach was not detected as a result of overlap with the pump and probe low photon flux between 650 and 700 nm. The signal relates to the transient broadening of the Au LSPR absorption band as a result of light excitation. Excitation of Au nanoparticles at 800 nm did not lead to electron injection into TiO_2_ as demonstrated by ultrafast transient infrared absorption spectroscopy (TIRAS) measurements, contrasting with what was observed when the system was excited at 550 nm (Figure S3 of the Supporting Information). At that excitation wavelength, there is an apparent positive infrared absorption related to hot electron injection into the TiO_2_ conduction band from Au LSPR excitation.^30,31^

Power-dependent transient photoluminescence experiments exciting at 800 nm (Figure 7c and Figure S4 of the Supporting Information) revealed a cubic dependence of the signal, suggesting the involvement of three photons in the upconversion process. This contrasts with the linear dependence of the emission at 550 nm observed when the system was excited at 400 nm, as we have reported in a previous publication.^26^ The cubic dependence also favors an upconversion process rather than a nonlinear optical process, such as Raman scattering. Hyper Raman and coherent anti-Stokes Raman commonly have a quadratic dependency with the incoming light rather than a cubic dependency.^32^

Semiconductor photon upconversion mediated by plasmonic hot carrier injection has been theoretically suggested^33^ but remains to be demonstrated experimentally. In the proposed process, the excitation of LSPR on a metal can result in the injection of electrons and holes (hot carriers) into semiconductor conduction and valence bands, respectively, with suitable energy levels. The electrons and holes can radiatively recombine either on the accepting semiconductor or after being transferred to another. The second semiconductor improves charge separation and, thus, process efficiency but reduces the energy of the upconverted photon. The proposed mechanism cannot justify the process detected herein because transient absorption measurements ruled out the involvement of hot carrier injections; i.e., the approach reported within is purely photonic without hot carriers.

**Figure 8:**
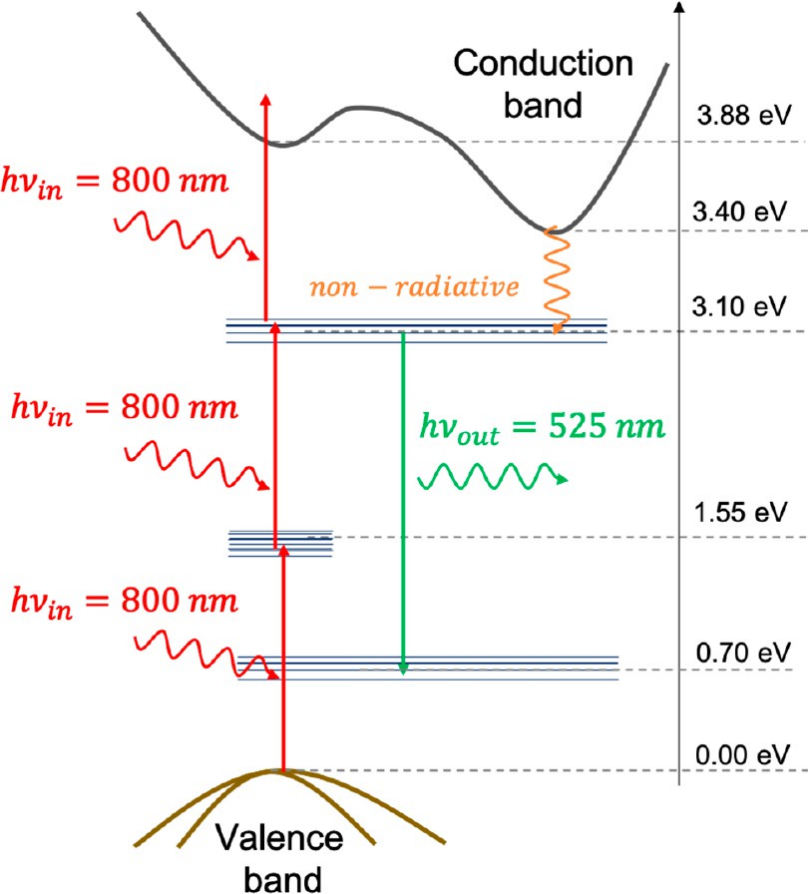
Level diagram describing the mechanism responsible for three-photon upconversion on TiO_2_ involving three consecutive photon excitations of an electron from the valence band to the conduction band.

Figure 8 shows the potential mechanisms that are thought to be at play in the TiO_2_ upconversion process. The power-dependent measurements revealed that three photons are involved in the process. A logical mechanism is to consider electron excitation from the valence band to the conduction band using the intraband trap states (Figure 8), where the first photon brings the electron into the 800 nm hole trap state, the second photon brings the electron into the 400 nm electron trap state, and the third photon brings the electron into the conduction band, a process commonly called ladder climbing.^34^ Once in the conduction band, the electron is allowed to relax into the trap state and recombine with a shallow trap hole, resulting in the emission of green light. This process is prevalent in ultrafast pump–probe experiments, which use ultrashort and intense infrared pulses to reach electronic levels via virtual vibrational states.^35^ In the presence case, climbing the ladder is performed using actual states, which are long-lived compared to the virtual states, making the process probable at lower laser fluencies.

**Figure 9:**
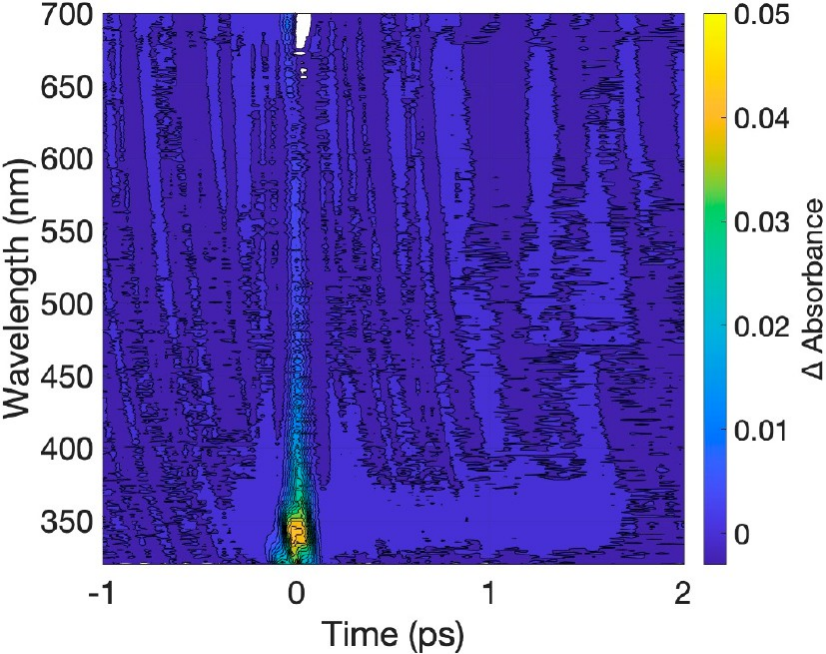
TAS of TiO_2_ excited at 800 nm and 18 mW pump laser fluence.

TAS measurements on TiO_2_ excited at 800 nm with different pump laser fluencies were performed to support the postulated mechanism. The results are shown in Figure 9 and Figure S5 of the Supporting Information. At high pump laser fluence (Figure 9), a nearly instantaneous positive signal around 320–350 nm (TiO_2_ conduction band edge) appears, which is also observed at a lower laser fluence (Figure S5 of the Supporting Information). According to the postulated hypothesis, an increase in TiO_2_ conduction band electron population is expected if the consecutive three-photon absorption promoting electrons from the valence to the conduction band via the gap trap states is the culprit; i.e., the observation supports the mechanism depicted in Figure 8.

**Figure 10:**
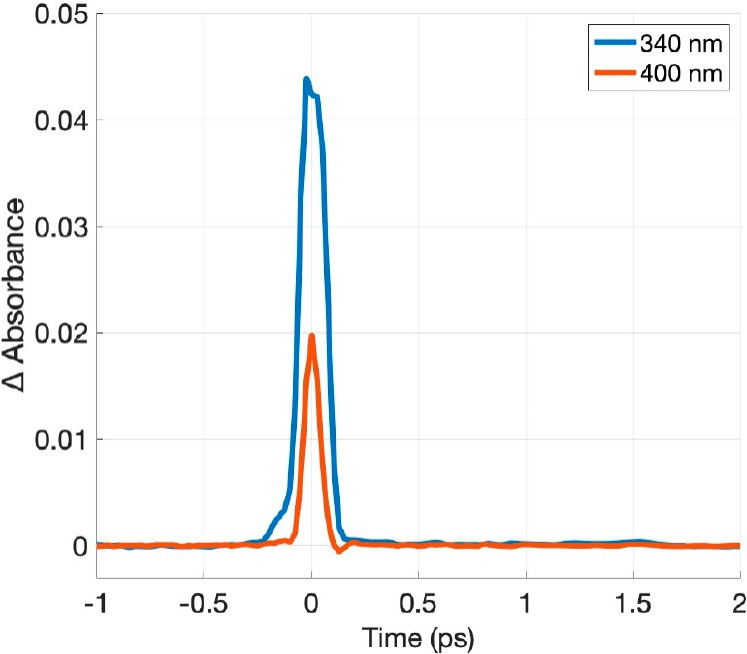
Kinetic traces extracted at 340 and 400 nm from the TAS contour plot of TiO_2_ excited at 800 nm and 18 mW pump laser fluence.

Further support for climbing the ladder mechanism can be found in the contour plot and kinetic traces in Figure 10. Part of the excited electron population in the TiO_2_ conduction band relaxes rapidily into the electron trap state centered at 400 nm (Figure 10), which is faster than our temporal resolution. However, this could also be ascribed to the filling of the 400 nm state, which occurs in both proposed mechanisms. Once there, part of the electrons recombines radiatively with a hole located at 680–700 nm. The hole state filling with electrons is noticeable on the TAS contour map measured with higher laser pump fluence. The entire process occurs within 1 ps, explaining the ultrafast nature of the emitted upconversion photons detected in the transient photoluminescence studies shown in Figure 7b.

The TAS experiments highlight two pertinent aspects. First, are the real states detected within the bandgap of TiO_2_ involved in the upconversion process? The ultrafast population of the conduction band with the electrons promoted via three-photon absorption and their fast relaxation could also be rationalized using virtual states as process mediators. To test this hypothesis, similar experiments were carried out using ZnO instead of TiO_2_. ZnO has a matching bandgap and is a strong emitter in the green but does not have trap states localized at 1.55 eV (800 nm) and 3.1 eV (400 nm) from the edge of the valence band.^36^ In this case, no upconverted signal was detected, corroborating the importance of TiO_2_ trap states for the process.

The second important aspect is the significant difference in the photoluminescence lifetime between the sample excited at 320 nm (bandgap excitation) and via three-photon absorption at 800 nm. Excitation at 320 nm is significantly more efficient, and consequently, many electron–hole pairs are formed, leading to a higher emission quantum yield. The temporal profile of this emission contains the short-lived emission related to fast trap filling (quick relaxation) and the long-lived emission connected to charge recombination, which took longer to find an available trap state. When there is a significant number of excited electrons and a limited number of trap states are available, the trap filling can take hundreds of picoseconds and even nanoeconds.^37^ This will “delay” the emission, consistent with what was observed in Figure 3. In the case of three-photon upconversion, only a few electrons are promoted in comparison to the number of available trap states. Consequently, only an ultrafast photoluminescence signal is present.

The proposed mechanisms are purely photonic, where the plasmonic gold nanoparticles enhance the optical absorption of the TiO_2_ transitions.^38,39^ This is distinct from the upconversion process via triplet–triplet annihilation in molecules and lanthanide emission through energy migration in hybrid materials. The most phenomenologically relatable system is the two-level single organolead halide perovskite nanocrystal in a resonance-adjustable plasmonic nanocavity reported by Lu et al.^19^ Their upconversion plasmonic nanolaser system utilizes a near-infrared pump laser that excites electron–hole pairs in the perovskite nanocrystal through two-photon absorption. The radiative recombination of relaxed electron–hole pairs emits energy quanta at visible wavelengths, which are then transferred to modes of the plasmonic cavity with adjustable plasmon resonance. The process has a very low lasing threshold at a cryogenic temperature (ca. 6 K), making it suitable for laser applications.

The system proposed herein upconverts near-infrared light using existing trap states within the TiO_2_ bandgap. The cryogenic temperature enhances the photoluminescence signal by prolonging the lifetime of the excited state. However and in contrast with the finding by Lu et al.,^19^ the enhancement is not dramatic because the process quantum yield is primarily affected by the electron occupancy of the trap state at 800 nm from the valence band rather than the lifetime of the excited state, which is physical and not virtual like in the case of the experiment by Lu et al.^19^ The advantage of the proposed system is that it can operate at room temperature. Its quantum yield can be increased by improving material emissivity, i.e., by material engineering, because the low emissivity of TiO_2_ is the primary bottleneck.^40^ Still, the semiconductor provided the scientific basis for a novel upconversion mechanism that relaxes on the ultrafast time scale, enabling it to outcompete thermal relaxation.

In conclusion, a novel three-photon mechanism for infrared-to-visible light was observed in TiO_2_ solid films. The process involves three photons and the use of the intraband electronic states. The efficiency of the process can be significant if the semiconductor system is coupled to plasmonic nanoparticles. The process can be further improved with better emitting wide bandgap semiconductors with intraband trap holes and electron states with suitable energy levels. The finding can pave the way for development of upconversion solid plasmon/semiconductor systems. Additionally and from a longer perspective, because the process relies on pre-existing trap states, one might consider other possibilities involving even more photons and lower energy transitions. This would depend upon only our ability to create such materials rather than the photonic process itself.

## Supporting Information Available

Additional data to support the main results that are presented herein (PDF)
Transparent Peer Review report available (PDF)
