# Supplementary material for: Ultrafast Infrared-to-Visible Photon Upconversion on Plasmon/TiO2 Solid Films
Source: J Phys Chem Lett. 2023 Jun 30;14(27):6255–62. doi: 10.1021/acs.jpclett.3c01208 (PMC10350964; doi:10.1021/acs.jpclett.3c01208)
Supplement: Supplementary file 1 — jz3c01208_si_001.pdf [file jz3c01208_si_001.pdf]

Supporting information

Ultrafast Infrared-to-Visible Photon Upconversion on Plasmon/TiO_2 Solid Films

Xianshao Zou[a,b*#], Robert Bericat Vadell[b#], Bin Cai[b], Xinjian Geng[b], Ananta Dey[b], Yawen Liu[b], Axel Gudmundsson[b], Jie Meng[c], and Jacinto Sá[b,d,e*]

[a] Qingdao Innovation and Development Base, Harbin Engineering University; Qingdao, 266 000, China.

[b] Physical Chemistry Division, Department of Chemistry - Angstrom Laboratory, Uppsala University, Box 523, 751 20 Uppsala, Sweden.

[c] Division of Chemical Physics, Lund University; Lund, 221 00, Sweden.

[d] Peafowl Plasmonics AB; Uppsala, 756 51, Sweden.

[e] Institute of Physical Chemistry, Polish Academy of Sciences, Marcina Kasprzaka 44/52, 01-224 Warsaw, Poland.

# equal contribution

Email: xianshao.zou@hrbeu.edu.cn; jacinto.sa@kemi.uu.se

Additional data

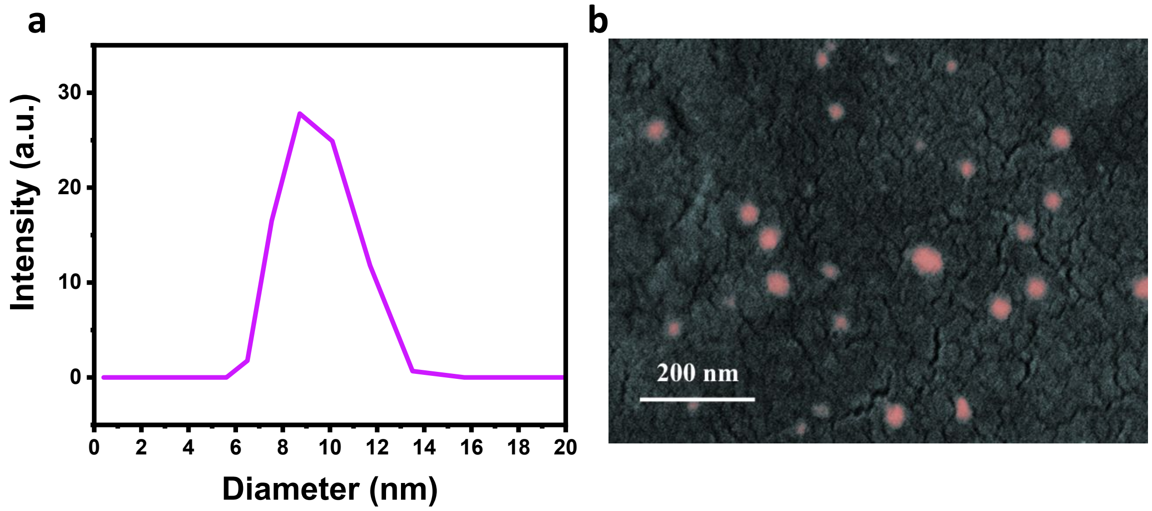

Figure S1. Au nanoparticles size. A) Dynamic light scattering (DLS), and B) Scanning electron microscopy (SEM) of Au nanoparticles on a glass slide.

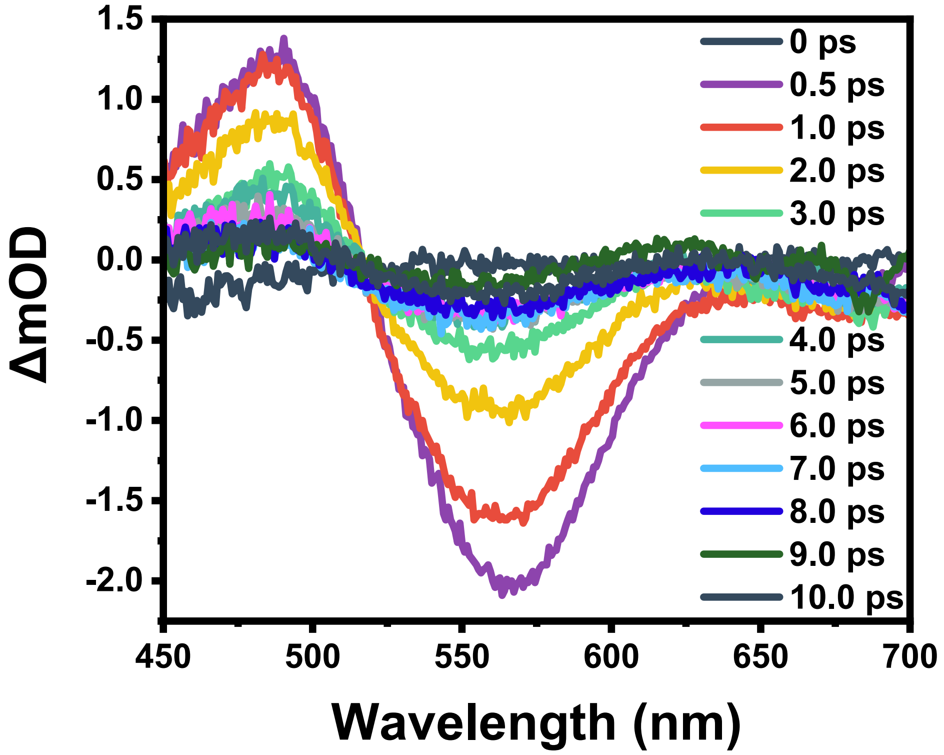

Figure S2. Transient absorption spectroscopy (TAS) of Au/TiO_2 excited at 800 nm.

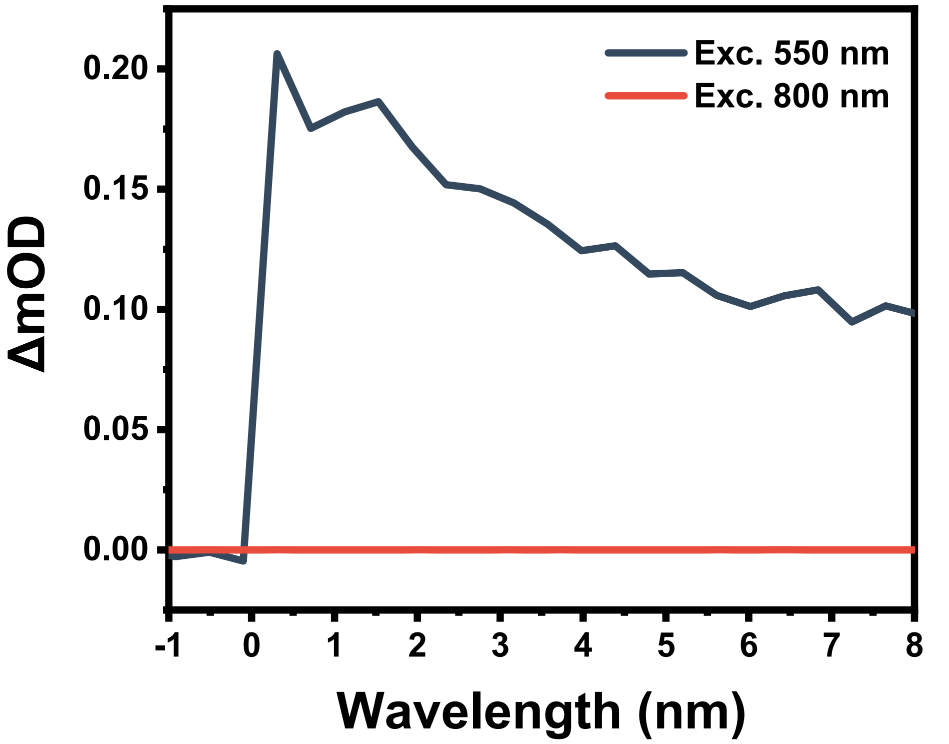

Figure S3. Kinetic trace extracted at 4600 nm (0.27 eV) from the transient infrared absorption spectroscopy (TIRAS) of Au/TiO_2.

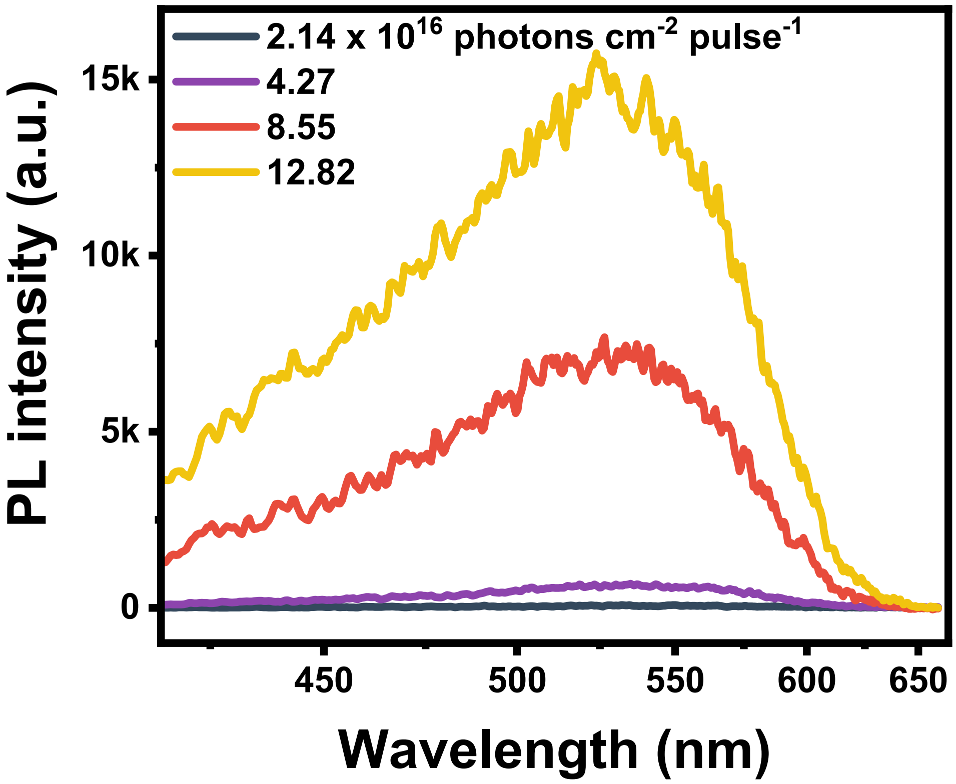

Figure S4. Cumulative emission signal of Au/TiO_2 excited at 800 nm at different laser fluencies.

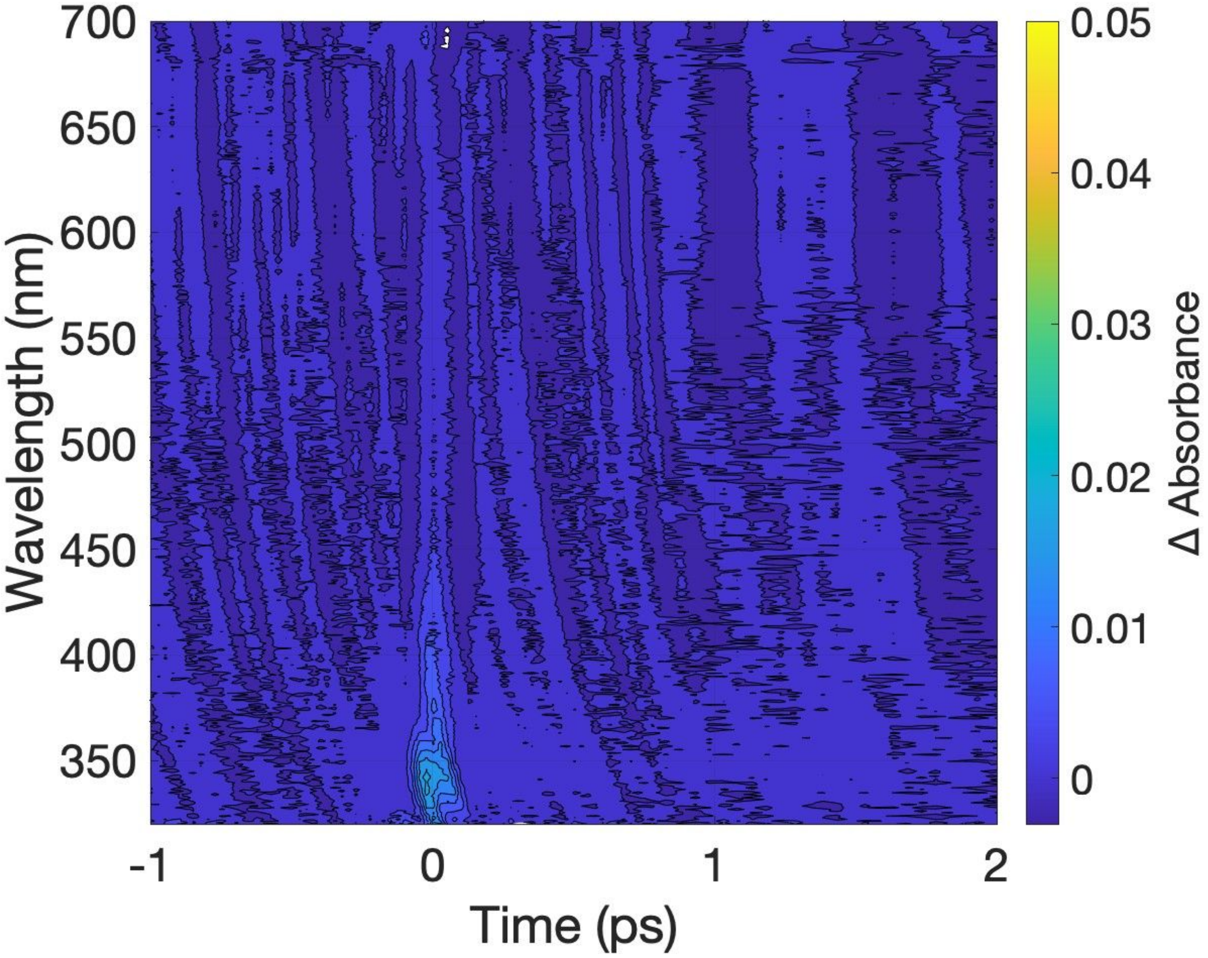

Figure S5. Transient absorption spectroscopy (TAS) of TiO_2 excited at 800 nm and 9 mW pump laser fluence.

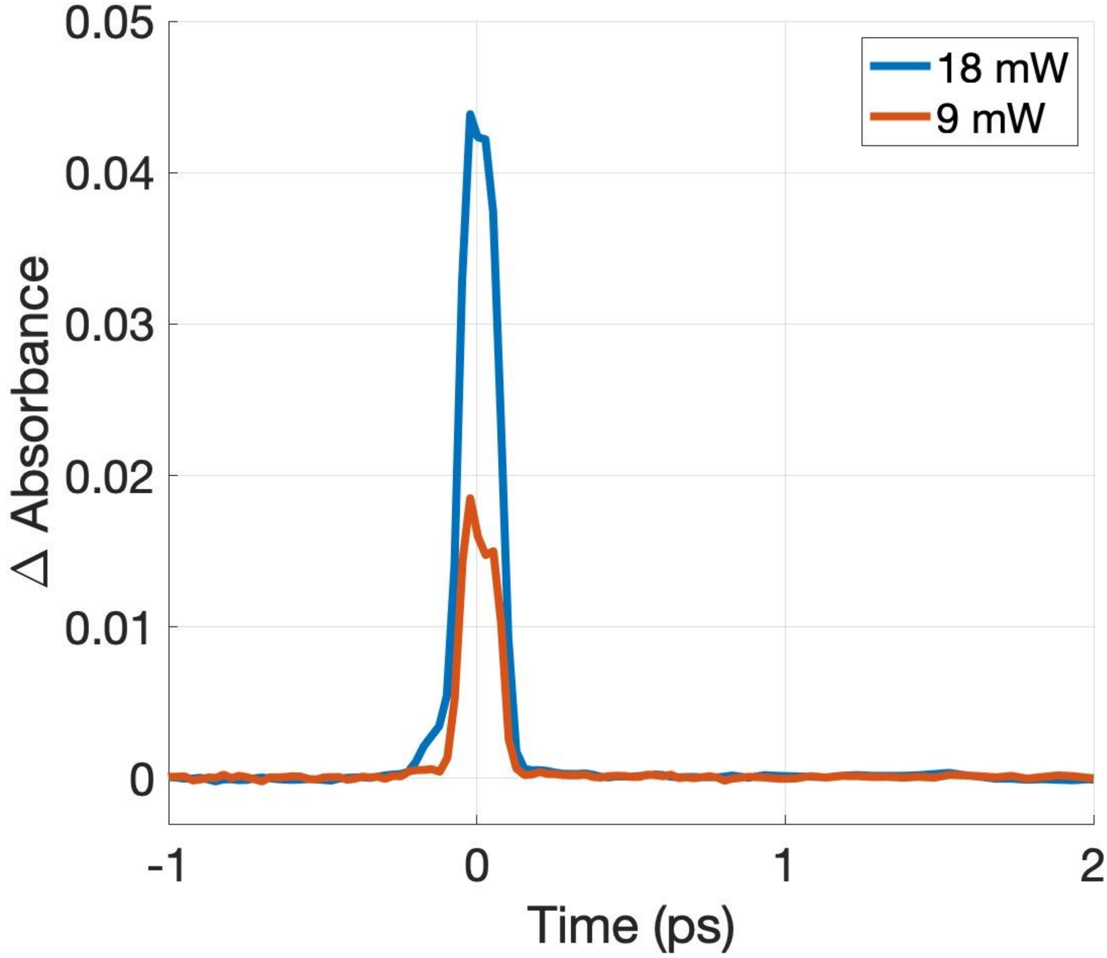

Figure S6. Comparison of the kinetic trace extracted at 340 mm from the TAS of TiO_2 excited at 800 nm with different laser fluencies.

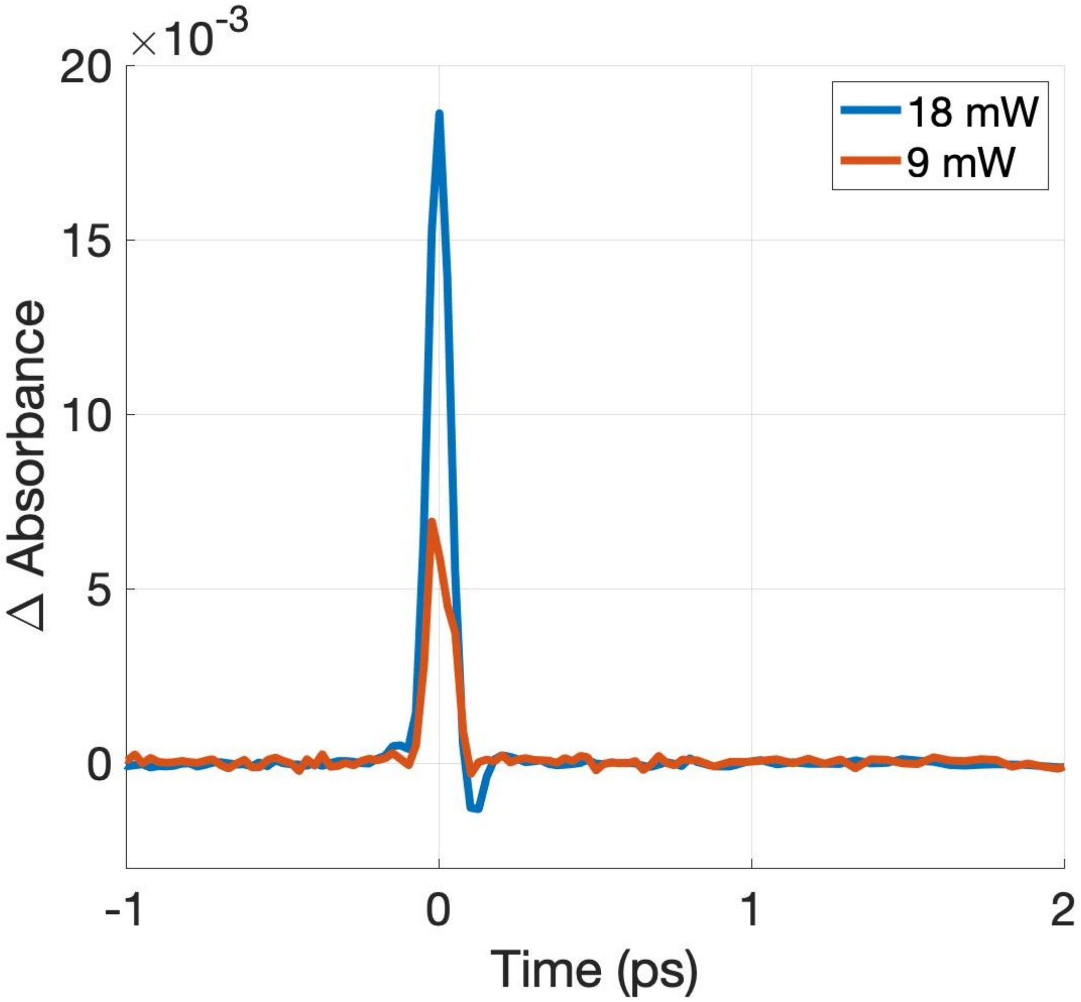

Figure S7. Comparison of the kinetic trace extracted at 400 mm from the TAS of TiO_2 excited at 800 nm with different laser fluencies.
