# Supplementary material for: Ultrafast Infrared-to-Visible Photon Upconversion on Plasmon/TiO2 Solid Films
Source: J Phys Chem Lett. 2023 Jun 30;14(27):6255–62. doi: 10.1021/acs.jpclett.3c01208 (PMC10350964; doi:10.1021/acs.jpclett.3c01208)
Supplement: Supplementary file 2 — jz3c01208_si_002.pdf [file jz3c01208_si_002.pdf]

jz-2023-01208g.R1

Name: Peer Review Information for "Ultrafast Infrared-to-Visible Photon Upconversion on Plasmon/TiO2 Solid Films"

First Round of Reviewer Comments

Reviewer: 1

Comments to the Author

The manuscript entitled "Ultrafast Infrared-to-Visible Photon Upconversion on Plasmon/TiO2 Solid Films" reports a new mechanism by taking advantage of TiO2 intra-bandgap energy state to perform photon upconversion. As acknowledged in the introduction, this study holds notable research significance. However, there are still notable deficiencies in experimental design and the elucidation of the mechanism in the article. The following recommendations are provided:

1. The authors extensively emphasize in the introduction the prevention of infrared radiation by building glass. However, they provide limited discussion on the current state of research in this area, with only a brief reference to the work by Lu et al. on upconversion plasmonic lasing. This insufficiency in substantiating the research background undermines the scholarly strength of the article. Also, the author should expand the discussion of photon upcoversion via lanthanide-doped inorganic nanoparticles, which is an important area of research (e.g.; triplet excited state stabilization, see Han, S. et al. Nature 2020, 587, 594–599; Nature Communications 2021, 12, 3704).

2. In Figure 7.b, it is recommended to select an appropriate testing time window to avoid the omission of fast process signals due to inadequate temporal resolution. Alternatively, when plotting the graph, it would be advisable to exclude the segments after the disappearance of the signal.

3. The assertions made in the article regarding the two potential mechanisms discussed in Figure 8 and Figure 9 lack adequate substantiation based on the presented evidence. To strengthen the claims, it is advisable to conduct further transient absorption tests, specifically observing the ultrafast formation process of the excited states at the picosecond timescale preferably at different temperatures. This would provide valuable insights for distinguishing between the two proposed mechanisms.

4. Additionally, there are certain instances in the manuscript where wording can be further improved. For instance, in the abstract, there is redundant phrasing in the statement "no charge injection or change in TiO2 photoluminescence TiO2 decay." It is advised to revise these sentences to enhance clarity and precision. Also, in Figure S3, the x-axis should be "Time(ps)" rather than "Wavelength(nm)".

Reviewer: 2

Comments to the Author

The authors present a series of measurements performed on a TiO_2 film overcoated with a layer of Au nanoparticles. The authors find that when the film is illuminated by an 800 nm laser source, the film produces upconverted emission centered near 525 nm. The intensity of this upconverted emission is enhanced by 20 times by the presence of gold nanoparticles. Interestingly, the authors find the upconverted emission shows a cubic dependence on the excitation power, which leads the authors to propose a unique, sequential 3 photon absorption scheme, summarized by figure 9, to explain their data.

Unfortunately, in reading this manuscript I have found it suffers from several significant deficiencies. As I argue below, the scientific picture the authors present to rationalize their results in figure 9 is insufficient. Moreover, the motivation provided for the work, that of developing better window treatments for mediating the influx of solar heat into buildings, is weak and somewhat misleading. The manuscript also suffers from several typos that suggest it was subjected to only a cursory level of polishing prior to its submission. For example, figure 1 contains two versions of its a and b panels and "Ultrafast" in the manuscript's title is misspelled.

In its present form, this manuscript does not provide fundamental insight into the upconversion behavior of the TiO_2:Au system the authors present. As such, I find it is not suitable for publication by *J. Phys. Chem. Lett.* I recommend that this manuscript be rejected and significantly revised before submission to a different journal. In revising this manuscript, the authors must, at a minimum, address the following points:

1) The authors postulate that population of a hole trap state by an 800 nm photon followed by sequential excitation of an electron from this state produces a state that can emit light a 525 nm (figure 9). However, the authors' data does not support this mechanism. In the scheme illustrated in figure 9, only 2 photons are ultimately responsible for producing emission at 525 nm. This should strictly give rise to a quadratic, rather than a cubic, scaling with the incident photon flux. The authors argue that additional population placed in the hole trap state at 1.55 eV should increase the efficiency of upconversion, which is true, but this should not disrupt the quadratic scaling of the process unless the interaction of pairs charge carriers in the trap state is fundamental to the upconversion process.

2) The authors do not comment at all on the lifetime of the emission observed when exciting TiO_2 using different excitation wavelengths. Exciting the system at 320 nm moves a charge from TiO_2's valence to its conduction band and gives rise to emission bands peaked at ~500 nm and ~1000 nm that persist for several 10s of picoseconds (figure 3). In sharp contrast, exciting the system at either 400 nm or 800 nm produces a short burst of emission that appears to decay in ~1-2 ps following excitation (figures 4 & 6). I suspect this short emission may track the instrument response function of the streak camera used to collect time-resolved emission (an instrument response function is not provided), which means its lifetime could be even shorter. Emission produced by exciting at 400 nm or 800 nm also appears to be spectrally shifted, peaking at ~545 nm and no evidence of emission at ~1000 nm is provided.

   These differences suggest the states involved in producing emission when exciting the system at 400 nm or 800 nm are fundamentally different than those that produce emission when exciting the system at 320 nm. 320 nm excitation produces electron-hole pairs that trap and recombine to produce emission at ~500 nm and ~1000 nm. The fact that a finite lifetime is seen for this emission supports this assigned mechanism.

On the other hand, the observation that emission generated when exciting the system at 400 nm or 800 nm is emitted nearly instantaneously, suggests it is instead produced by a nonlinear optical process that does not excite carriers to a high-energy state. Processes such as electronic Raman scattering would give rise to emission without producing an electronically excited state. Electronic Raman scattering should also be enhanced by the presence of Au, and depending on the pathway that leads to this scattering, it could display a cubic dependence on the excitation power. The fact that there are several defect states in the TiO_2 could also lead to resonance enhancement of this scattering that could produce the shift in emission wavelength seen when exciting the sample at 800 nm/400 nm vs. 320 nm and also explain why no emission at ~1000 nm is seen when exciting the system at 800 nm/400 nm, which would be expected if emission was coming from charge carriers relaxing through a manifold of trap states.

What is the reason that the authors feel confident in ruling out emission pathways involving Raman scattering or other nonlinear processes that do not produce states with finite lifetimes?

3) A pump probe experiment that probes the formation of induced absorption bands associated with photoexcited charge carriers could be useful in better assigning the origin of the dynamics the authors report. In particular, a pump, repump, probe experiment that uses a pair of 800 nm pulses, separated in time, to excite the sample would allow the authors to discern if charge carriers placed into a hole defect state are indeed responsible for emission produced at 545 nm when exciting the system at 800 nm.

4) The authors motivate their work by stating the upconversion material they report could be used as a window coating that would reject solar infrared radiation by converting it into visible light, thereby allowing better management of a building's internal environment. This motivation is fraught on several levels. (1) By converting infrared light into visible radiation, no energy is actually being rejected. It's just being radiated into the building at a different color. (2) The authors find that when they excite the system using 300 fs pulses that contain 2.14×10^{16} photons/cm^2, they obtain negligible emission (figure S4). Averaged over the pulse duration, this corresponds to an incident photon flux of ~7.1×10^{13} photons/cm^2*fs. At 800 nm, the solar irradiance at Earth's surface is about 1 W/m^2, which translates to 0.4 photons/cm^2*fs, which is ~14 orders of magnitude smaller. This suggests that upconversion produced by this system is likely to be irrelevant under solar flux. (3) I doubt that most workers would find it comfortable to work in a building whose windows luminesced green light.

   I simply don't see a potential market for using this technology as a window coating for energy management. Using that concept to motivate the work seems disingenuous and I would urge the authors to consider motivating their work in a different way.

5) The manuscript contains several typos and some of the language it employs is unclear. A few notable issues in need of correction are listed below, but this list is not exhaustive:

   a. "Ultrafast" is misspelled in the title
   b. Figure 1 contains two versions of panels a and b.
   c. It is unclear what a "winglet" is in the description of data contained in figure S2 (p. 4)
   d. On p. 4, "power-dependence transient photoluminescence" should be "power-dependent transient photoluminescence"
   e. On p. 2, "heating getting into the building" should be "heat getting into the building"
   f. On p. 2, it is unclear what a "listed building" is.

g. A citation for peak assignments made in Figure 1b is missing.

6) I find the images in figure 7d – 7g to not be useful. In particular, it is unclear if any upconversion emission is seen at all in the images recorded at 295 K. I recommend removing panels e and g from this figure.

Author's Response to Peer Review Comments:

Answers to the Reviewers' comments

Reviewer: 1
The manuscript entitled "Ultrafast Infrared-to-Visible Photon Upconversion on Plasmon/TiO2 Solid Films" reports a new mechanism by taking advantage of TiO2 intra-bandgap energy state to perform photon upconversion. As acknowledged in the introduction, this study holds notable research significance. However, there are still notable deficiencies in experimental design and the elucidation of the mechanism in the article.
We commend the Reviewer for acknowledging the manuscript's novelty and scientific merit. We thank the Reviewer for the time dedicated to the revision of the manuscript and the precious recommendations. We tried within our experimental capabilities to provide additional supporting information. Furthermore, we addressed all the comments below and updated the manuscript accordingly. We hope the revised version suppresses the initial reservations that the Reviewer added.

The following recommendations are provided:

1. The authors extensively emphasize in the introduction the prevention of infrared radiation by building glass. However, they provide limited discussion on the current state of research in this area, with only a brief reference to the work by Lu et al. on upconversion plasmonic lasing. This insufficiency in substantiating the research background undermines the scholarly strength of the article. Also, the author should expand the discussion of photon upcoversion via lanthanide-doped inorganic nanoparticles, which is an important area of research (e.g.; triplet excited state stabilization, see Han, S. et al. Nature 2020, 587, 594–599; Nature Communications 2021, 12, 3704).
We thank the Reviewer for bringing the lanthanide-doped developments to our attention. This exciting technological platform for photon upconversion suppresses some of the issues with molecular systems, namely the fact they are materials (easy-to-make devices) and are chemically more stable. We added a paragraph to the introduction on this and the respective references. We added paragraph on molecular triple-triple annihilation to cover the developments in converting the system into materials. We have also increased the discussion to contextualize the findings of the work concerning the field. However, we focused on the comparison with the work published by Lu et al. because it is phenomenologically similar to ours, namely a pure photonic process.

2. In Figure 7.b, it is recommended to select an appropriate testing time window to avoid the omission of fast process signals due to inadequate temporal resolution. Alternatively, when plotting the graph, it would be advisable to exclude the segments after the disappearance of the signal.
We thank the Reviewer for this comment. Figure 7b x-axis has been adjusted to an appropriate time window.

3. The assertions made in the article regarding the two potential mechanisms discussed in Figure 8 and Figure 9 lack adequate substantiation based on the presented evidence. To strengthen the claims, it is advisable to conduct further transient absorption tests, specifically observing the ultrafast formation process of the excited states at the picosecond timescale preferably at different temperatures. This would provide valuable insights for distinguishing between the two proposed mechanisms.
We thank the Reviewer for this comment. We added to the manuscript the transient absorption spectroscopy (TAS) data for TiO2 excited at 800 nm with different laser fluencies (new figures 10, 11 and S5). At high laser fluence, a nearly instantaneous positive signal around 320-350 nm (TiO2 conduction band edge) appears also observed at a lower laser fluence (figure S5). This observation suggests consecutive three-photon absorption leading to a promotion of electrons from the valence via the gap trap states to the conduction band, which supports the mechanism

proposed in Figure 8. The findings change our initial assumption and thus we thank once more the Reviewer for suggestion this measurement.
We added to the text:
TAS measurements on TiO_2 excited at 800 nm with different pump laser fluencies were performed to establish which of the mechanisms is prevalent. The results are shown in figures 10 and S5. At high pump laser fluence (figure 10), a nearly instantaneous positive signal around 320-350 nm (TiO_2 conduction band edge) appears, which is also observed at a lower laser fluence (figure S5). According to the postulated hypotheses, an increase in TiO_2 conduction band electron population is expected if the consecutive three-photon absorption promoting electrons from the valence to the conduction band via the gap trap states is the culprit, i.e., the observation supports the mechanism depicted in Figure 8.
Further support for climbing the ladder mechanism can be found in the contour plot and kinetic traces in figure 10 and 11, respectively. Part of the excited electrons population in the TiO2 conduction band relaxes into the electron trap state centred at 400 nm within 100-200 fs, clearly seen in the kinetic trace analysis (figure 11). Once there, part of the electrons recombines radiatively with a hole located at 680-700 nm. The hole state filling with electrons is noticeable on the TAS contour map measured with higher laser pump fluence. The entire process occurs within 1 ps, explaining the ultrafast nature of the emitted upconversion photons.
We cannot perform TAS experiments under cryogenic temperature in our setup, which we agree would help substantiate if the mechanism proposed in figure 9 can also be present. TAS experiments with the Au/TiO2 excited at 550 nm (peak of Au plasmon resonance) and 800 nm (Figure S2) are entirely dominated by the Au plasmon resonance signal, making it challenging to see TiO2 signals below 10 ps.

4. Additionally, there are certain instances in the manuscript where wording can be further improved. For instance, in the abstract, there is redundant phrasing in the statement "no charge injection or change in TiO2 photoluminescence TiO2 decay." It is advised to revise these sentences to enhance clarity and precision. Also, in Figure S3, the x-axis should be “Time(ps)” rather than “Wavelength(nm)”.
We thank the Reviewer for bringing this to our attention. We performed a detailed English check using software and the authors' abilities. We think the revised version is clean of typos and English inconsistencies.

Reviewer: 2
The authors present a series of measurements performed on a TiO2 film overcoated with a layer of Au nanoparticles. The authors find that when the film is illuminated by an 800 nm laser source, the film produces upconverted emission centered near 525 nm. The intensity of this upconverted emission is enhanced by 20 times by the presence of gold nanoparticles. Interestingly, the authors find the upconverted emission shows a cubic dependence on the excitation power, which leads the authors to propose a unique, sequential 3 photon absorption scheme, summarized by figure 9, to explain their data. Unfortunately, in reading this manuscript I have found it suffers from several significant deficiencies. As I argue below, the scientific picture the authors present to rationalize their results in figure 9 is insufficient. Moreover, the motivation provided for the work, that of developing beter window treatments for mediating the influx of solar heat into buildings, is weak and somewhat misleading. The manuscript also suffers from several typos that suggest it was subjected to only a cursory level of polishing prior to its submission. For example, figure 1 contains two versions of its a and b panels and “Ultrafast” in the manuscript’s title is misspelled. In its present form, this manuscript does not provide fundamental insight into the upconversion behavior of the TiO2:Au system the authors present. As such, I find it is not suitable for publication by *J. Phys. Chem. Lett.* I recommend that this manuscript be rejected and significantly revised before submission to a different journal.
We thank the Reviewer for the time dedicated to the revision of the manuscript and the precious recommendations. We tried within our experimental capabilities to provide additional supporting

information for the proposed mechanism. Furthermore, we addressed all the comments below and updated the manuscript accordingly. We hope the revised version suppresses the initial reservations that the Reviewer added. We want to mention that the potential application, even if further substantiated, can only be considered if the process quantum yield is improved. Therefore, the manuscript's merits are on the mechanism novelty for photon upconversion rather than the potential application.

In revising this manuscript, the authors must, at a minimum, address the following points:
1. The authors postulate that population of a hole trap state by an 800 nm photon followed by sequential excitation of an electron from this state produces a state that can emit light a 525 nm (figure 9). However, the authors' data does not support this mechanism. In the scheme illustrated in figure 9, only 2 photons are ultimately responsible for producing emission at 525 nm. This should strictly give rise to a quadratic, rather than a cubic, scaling with the incident photon flux. The authors argue that additional population placed in the hole trap state at 1.55 eV should increase the efficiency of upconversion, which is true, but this should not disrupt the quadratic scaling of the process unless the interaction of pairs charge carriers in the trap state is fundamental to the upconversion process.
We thank the Reviewer for this comment. In Figure 9, there is an evident two-photon excitation leading to a promotion of two electrons into the hole trap state at 800 nm, followed by a single photon excitation from electrons in the 800 nm trap state to the 400 nm electron trap, from which the charge emits the 525 nm photon. This process involves 3-photons via two different trap states. We re-worded the text to make this crystal clear.

2. The authors do not comment at all on the lifetime of the emission observed when exciting TiO2 using different excitation wavelengths. Exciting the system at 320 nm moves a charge from TiO2's valence to its conduction band and gives rise to emission bands peaked at ~500 nm and ~1000 nm that persist for several 10s of picoseconds (figure 3). In sharp contrast, exciting the system at either 400 nm or 800 nm produces a short burst of emission that appears to decay in ~1-2 ps following excitation (figures 4 & 6). I suspect this short emission may track the instrument response function of the streak camera used to collect time-resolved emission (an instrument response function is not provided), which means its lifetime could be even shorter. Emission produced by exciting at 400 nm or 800 nm also appears to be spectrally shifted, peaking at ~545 nm and no evidence of emission at ~1000 nm is provided.
These differences suggest the states involved in producing emission when exciting the system at 400 nm or 800 nm are fundamentally different than those that produce emission when exciting the system at 320 nm. 320 nm excitation produces electron-hole pairs that trap and recombine to produce emission at ~500 nm and ~1000 nm. The fact that a finite life□me is seen for this emission supports this assigned mechanism.
On the other hand, the observation that emission generated when exciting the system at 400 nm or 800 nm is emited nearly instantaneously, suggests it is instead produced by a nonlinear optical process that does not excite carriers to a high-energy state. Processes such as electronic Raman scatering would give rise to emission without producing an electronically excited state. Electronic Raman scatering should also be enhanced by the presence of Au, and depending on the pathway that leads to this scatering, it could display a cubic dependence on the excitation power. The fact that there are several defect states in the TiO2 could also lead to resonance enhancement of this scatering that could produce the shift in emission wavelength seen when exciting the sample at 800 nm/400 nm vs. 320 nm and also explain why no emission at ~1000 nm is seen when exciting the system at 800 nm/400 nm, which would be expected if emission was coming from charge carriers relaxing through a manifold of trap states. What is the reason that the authors feel confident in ruling out emission pathways involving Raman scatering or other nonlinear processes that do not produce states with finite lifetimes?
We thank the Reviewer for this comment. Detailed analysis of the photophysical behaviour of TiO2 and Au/TiO2 after TiO2 bandgap excitation was the central theme of a recently published paper from our group. And therefore, we added to the text:

Detailed photophysical study of TiO_2 and Au/TiO_2 after TiO_2 bandgap excitation was performed in our previous publication,[REF1] and its therefore outside this contribution. Howevre, the kinetic traces analysis presented in Figure 3b is consistent with what has been measured before but as it will be shown very different from what happens when exciting below the band gap energy.
[REF1] Zou, X.; Vadell, R. V.; Liu, Y.; Mendalz, A.; Drillet, M.; Sá, J. Photophysical Study of Electron and Hole Trapping in TiO_2 and TiO_2/ Au Nanoparticles through a Selective Electron Injection. *J. Phys. Chem. C* **2022**, *126*, 21467-21475.
We think what is important to stress is these dynamics are consistent with other studies but completely different from what we saw when exciting below the TiO2 bandgap energy. We added to the text:
Analysis of the time component of the emission centred at 550 nm revealed an ultrafast radiative relaxation that ceases within 10 ps, independent of the excitation wavelength being at 400 or 800 nm. The streak-camera instrument response of about 5-7 ps, precludes deeper analysis of the temporal response of the signal. However, it suggests a mechanism in the ultrafast domain compatible with multi-photon involvement mediated by real rather than virtual trap states.
As the Reviewer rightly suggests the ultrafast relaxation of the process could be interpreted as a nonlinear optical process that does not excite carriers to a high-energy state, such as electronic Raman scatering. However, Hyper Raman and Coherent Anti-Stokes Raman (Borman et al. Anal. Chem. 54 (1982) 1022) have a quandratic dependence and as far as we are aware there are no publications suggesting otherwise. Addtionally, the signal is too broad in energy to be related to Raman.
To establish which of the mechanisms proposed is prevalent, we performed transient absorption spectroscopy (TAS) data for TiO2 excited at 800 nm with different laser fluencies (new figures 10 and S5). We added to the manuscript the transient absorption spectroscopy (TAS) data for TiO2 excited at 800 nm with different laser fluencies (new figures 10, 11 and S5). At high laser fluence, a nearly instantaneous positive signal around 320-350 nm (TiO2 conduction band edge) appears also observed at a lower laser fluence (figure S5). This observation suggests consecutive three-photon absorption leading to a promotion of electrons from the valence via the gap trap states to the conduction band, which supports the mechanism proposed in Figure 8. The findings change our initial assumption and thus we thank once more the Reviewer for suggestion this measurement.
We added to the text:
TAS measurements on TiO_2 excited at 800 nm with different pump laser fluencies were performed to establish which of the mechanisms is prevalent. The results are shown in figures 10 and S5. At high pump laser fluence (figure 10), a nearly instantaneous positive signal around 320-350 nm (TiO_2 conduction band edge) appears, which is also observed at a lower laser fluence (figure S5). According to the postulated hypotheses, an increase in TiO_2 conduction band electron population is expected if the consecutive three-photon absorption promoting electrons from the valence to the conduction band via the gap trap states is the culprit, i.e., the observation supports the mechanism depicted in Figure 8.
Further support for climbing the ladder mechanism can be found in the contour plot and kinetic traces in figure 10 and 11, respectively. Part of the excited electrons population in the TiO2 conduction band relaxes into the electron trap state centred at 400 nm within 100-200 fs, clearly seen in the kinetic trace analysis (figure 11). Once there, part of the electrons recombines radiatively with a hole located at 680-700 nm. The hole state filling with electrons is noticeable on the TAS contour map measured with higher laser pump fluence. The entire process occurs within 1 ps, explaining the ultrafast nature of the emitted upconversion photons.

3. A pump probe experiment that probes the formation of induced absorption bands associated with photoexcited charge carriers could be useful in beter assigning the origin of the dynamics the authors report. In particular, a pump, repump, probe experiment that uses a pair of 800 nm pulses, separated in time, to excite the sample would allow the authors to discern if charge carriers placed into a hole defect state are indeed responsible for emission produced at 545 nm when exciting the system at 800 nm.

We thank the Reviewer for this comment. We added transient absorption spectroscopy (TAS) data for TiO2 excited at 800 nm with different laser fluencies to the revised manuscript. Unfortunately, our system does not allow for pump-pump-probe experiments. The excitation at different fluencies enabled us to monitor the absorption of the 400 nm trap state and evaluate if there was evidence for 3-photon excitation leading to bandgap excitation, which would support the mechanism reported in Figure 8. We think the additional data and explanations substantiate the proposed mechanism. The findings were described in point 2.

4. The authors motivate their work by stating the upconversion material they report could be used as a window coating that would reject solar infrared radiation by converting it into visible light, thereby allowing beter management of a building's internal environment. This motivation is fraught on several levels. (1) By converting infrared light into visible radiation, no energy is actually being rejected. It's just being radiated into the building at a different color. (2) The authors find that when they excite the system using 300 fs pulses that contain 2.14×10^{16} photons/cm^2, they obtain negligible emission (figure S4). Averaged over the pulse duration, this corresponds to an incident photon flux of ~7.1×10^{13} photons/cm^2*fs. At 800 nm, the solar irradiance at Earth's surface is about 1 W/m^2, which translates to 0.4 photons/cm^2*fs, which is ~14 orders of magnitude smaller. This suggests that upconversion produced by this system is likely to be irrelevant under solar flux. (3) I doubt that most workers would find it comfortable to work in a building whose windows luminesced green light.
I simply don't see a potential market for using this technology as a window coating for energy management. Using that concept to motivate the work seems disingenuous and I would urge the authors to consider motivating their work in a different way.
We thank the Reviewer for this comment. The aim of the manuscript is to report this new three-photon upconversion process. The potential application is a future perspective that requires material development so the quantum yield is improved. This is now clearly stated in the abstract, namely:
The process utilizes pre-existing trap states within the semiconductor band gap and involves three-photon absorption. If its quantum yield is dramatically improved, the proposed photon upconversion mechanism will provide a low-cost, retrofittable and low-maintenance alternative to dynamic glazing used to reduce infrared entering buildings.
We are perfectly aware that this is not suitable for application in the current status. We never claimed that we wanted to reject the infrared but rather to upconvert it. This is why this would fall into a completely different category of glazing from the existing one.
On a side note, while we are many iterations from making such Active glazing, we are actively working with one of the top-three in volume glass manufacturers in the world to make this possible, demonstrating its commercial interest. Remember that we are not necessarily trying to utilize the upconverted photon but rather to convert the near-infrared photons into visible photons, and our process 'consumes' three near-infrared photons every time the process occurs, establishing a theoretical maximum at 33%. However, since we are using pre-existing trap states in the material rather than virtual states, there are possibilities for other schemes to be involved using even more photons and lower transition energies.

5.The manuscript contains several typos and some of the language it employs is unclear. A few notable issues in need of correction are listed below, but this list is not exhaustive:

a) "Ultrafast" is misspelled in the title
   This was corrected in the revised version of the manuscript.
b) Figure 1 contains two versions of panels a and b.
   This was corrected in the revised version of the manuscript.
c) it is unclear what a "winglet" is in the description of data contained in figure S2 (p. 4)
   This has been clarified in the revised version of the text, but winglet is a standard label for the positive signals on the sides of the LSPR bleach.
d) On p. 4, "power-dependence transient photoluminescence" should be "power-dependent transient photoluminescence"

This was corrected throughout the text in the revised version of the manuscript.

e) On p. 2, “heating getting into the building” should be “heat getting into the building”
This was corrected in the revised version of the manuscript.

f) On p. 2, it is unclear what a “listed building” is.
This was changed to protected in the revised version of the manuscript.

g) A citation for peak assignments made in Figure 1b is missing.
This was corrected in the revised version of the manuscript.

We performed a detailed English check using software and the authors' abilities. We think the revised version is clean of typos and English inconsistencies.

6. I find the images in figure 7d – 7g to not be useful. In particular, it is unclear if any upconversion emission is seen at all in the images recorded at 295 K. I recommend removing panels e and g from this figure.
We thank the Reviewer for this comment. We wanted to show that in the case of the Au/TiO2 sample, we could see a faint green emission even at room temperature, which is clearly different from the process proposed by Lu et al that only occurs at cryogenic temperatures. Unfortunately, the emission was too faint for the camera to capture a good picture showing the green colour, but the faint emitted light. We are happy to remove this if the editorial team suggests it.

jz-2023-01208g.R2

Name: Peer Review Information for "Ultrafast Infrared-to-Visible Photon Upconversion on Plasmon/TiO2 Solid Films"

Second Round of Reviewer Comments

Reviewer: 2

Comments to the Author

While the authors have worked to address some of the concerns I raised in my initial review, I still do not believe this manuscript is suitable for publication. The authors postulate a mechanism for the upconverted emission that is not consistent with their experimental data. Moreover, I still find the motivation for the work they provide, that of making window coatings to reject infrared light for building heat management, detracts from their work. Below, I raise specific points the authors must address if this work is to be made suitable for publication.

1) In point 1 of my initial review, I questioned why the mechanism in figure 9 would exhibit a cubic scaling with incident power rather than a quadratic scaling. The authors replied that the initial process of absorption is a two-photon absorption process that promotes two electrons to the trap state situated at ~1.55 eV. However, it seems strange to me that the same transition couldn't simply be driven by a one photon absorption process. I can't think of a simple reason why a two-photon absorption process to produce a doubly excited state would prove to be more strongly allowed than a corresponding one photon absorption process that would promote a single electron.

Rather than pressing this point further, since the authors have concluded that the scheme in figure 9 is not supported by their data, I strongly favor simply removing it from the text to simplify the manuscript's discussion.

2) In point 2 of my initial review, I questioned the involvement of the trap state formed following excitation of TiO2's conduction band in producing upconverted emission. My reason for raising this concern is that direct excitation of an electron from the TiO2 valence to conduction band using 320 nm photons produces a state with a finite lifetime (figure 3) whereas time-resolved data measured when exciting the system at 800 nm produces short-lived emission that tracked the instrument response of the streak camera used by the authors (figure 6). This difference in lifetime suggested that a nonlinear mixing process that does not involve population of a real state was responsible for the upconverted emission produced by 800 nm excitation.

In response to this comment, the authors performed transient absorption measurements using 800 nm photons as the pump, which shows that the transient signal produced by 800 nm excitation is incredibly short lived, persisting for only ~100 fs or so (figure 10). This short timescale likely tracks the instrument response function of the transient absorption setup employed by the authors. This short-lived response

is consistent with the scenario I postulated in my prior review, wherein virtual states that lack a finite lifetime are responsible for the upconversion observed when photoexciting the system at 800 nm.

It is strange to me then that the authors take the data in figure 10 to support the mechanism highlighted in figure 8, wherein an electron is promoted via 3 photon absorption, from the TiO2 valence to conduction band. Presumably, such an electron would give rise to a state with a finite lifetime as is seen in figure 3. Since this is not what the authors observe, this argues against the scenario pictured in figure 8.

At present, the authors provide no mechanism that explains the difference in emission lifetime observed when photoexciting the system at 320 nm vs. 800 nm.

3) In point 4 of my initial review, I questioned the potential utility of the material the authors report for use as a window coating for building heat management. Certainly, the efficiency of this material needs to be improved if the authors ever hope to employ it to upconvert solar radiation. However, that was not my primary concern in questioning the utility of this material. Rather, I don’t fundamentally understand how transforming incident solar radiation from one color to another acts to reject any incident energy from the sun. Following upconversion, visible photons produced from near infrared light can be radiated into the building, which wouldn’t change the incident flux of energy from the sun. Moreover, converting incident near infrared light into visible light will make the windows appear to glow, which if efficient enough, could prove quite disconcerting to workers.

If the authors insist on using this idea for motivating their work, they need to do a better job in explaining why upconversion would lower the energy incident into an office building. Perhaps it needs to be coupled with some type of waveguide that could more easily reflect visible photons away from the building, or maybe direct them to solar cells embedded at the edges of the glass that could use them for power generation, etc. However, as written, the motivation for developing this material comes across as nonsensical since it doesn’t reject energy, and detracts from the results the authors report.

Author's Response to Peer Review Comments:

Answers to the Reviewer's comments

Reviewer: 2

While the authors have worked to address some of the concerns I raised in my initial review, I still do not believe this manuscript is suitable for publication. The authors postulate a mechanism for the upconverted emission that is not consistent with their experimental data. Moreover, I still find the motivation for the work they provide, that of making window coatings to reject infrared light for building heat management, detracts from their work. Below, I raise specific points the authors must address if this work is to be made suitable for publication.

We commend the Reviewer for acknowledging the improvements performed to the original manuscript. We thank the Reviewer for the time dedicated to the revision of the manuscript and the precious recommendations. We modified the manuscript to be consistent with the data and per the Reviewer's suggestions. Furthermore, we addressed all the comments below. We hope the revised version suppresses the initial reservations that the Reviewer added.

1) In point 1 of my initial review, I questioned why the mechanism in figure 9 would exhibit a cubic scaling with incident power rather than a quadratic scaling. The authors replied that the initial process of absorption is a two-photon absorption process that promotes two electrons to the trap state situated at ~1.55 eV. However, it seems strange to me that the same transition couldn't simply be driven by a one photon absorption process. I can't think of a simple reason why a two-photon absorption process to produce a doubly excited state would prove to be more strongly allowed than a corresponding one photon absorption process that would promote a single electron. Rather than pressing this point further, since the authors have concluded that the scheme in figure 9 is not supported by their data, I strongly favor simply removing it from the text to simplify the manuscript's discussion.

We thank the Reviewer for the comment. As rightly mentioned, the data is more consistent with the three-consecutive photon absorption; therefore, we removed the alternative explanation from the manuscript as suggested.

2) In point 2 of my initial review, I questioned the involvement of the trap state formed following excitation of TiO2's conduction band in producing upconverted emission. My reason for raising this concern is that direct excitation of an electron from the TiO2 valence to conduction band using 320 nm photons produces a state with a finite lifetime (figure 3) whereas time-resolved data measured when exciting the system at 800 nm produces short-lived emission that tracked the instrument response of the streak camera used by the authors (figure 6). This difference in lifetime suggested that a nonlinear mixing process that does not involve population of a real state was responsible for the upconverted emission produced by 800 nm excitation.

In response to this comment, the authors performed transient absorption measurements using 800 nm photons as the pump, which shows that the transient signal produced by 800 nm excitation is incredibly short lived, persisting for only ~100 fs or so (figure 10). This short timescale likely tracks the instrument response function of the transient absorption setup employed by the authors. This short-lived response is consistent with the scenario I postulated in my prior review, wherein virtual states that lack a finite lifetime are responsible for the upconversion observed when photoexciting the system at 800 nm.

It is strange to me then that the authors take the data in figure 10 to support the mechanism highlighted in figure 8, wherein an electron is promoted via 3 photon absorption, from the TiO2 valence to conduction band. Presumably, such an electron would give rise to a state with a finite lifetime as is seen in figure 3. Since this is not what the authors observe, this argues against the scenario pictured in figure 8.

At present, the authors provide no mechanism that explains the difference in emission lifetime observed when photoexciting the system at 320 nm vs. 800 nm.

We thank the Reviewer for the comment. The Reviewer is correct in stating that the short-lived nature of the absorption when exciting the system with 800 nm would indicate virtual state involvement. We agree that such states might play a role, but we have identified the existence of states at 800 and 400 nm from the TiO2 valence band. Those states are real, and the 400 nm excitation resulted in emission at 525 nm, which suggests state involvement. Moreover, if these states were irrelevant to the process, we could replicate the findings with a more emissive semiconductor like ZnO. However, our experiments show that this is not the case, and thus, we think the state within the gap of TiO2 is indeed essential.

The second aspect relates to the difference in emission lifetime when excited at 320 nm and 800 nm. When excited at 320 nm, we have significantly more electron-hole pairs formed and, consequently, a lot more emission. The temporal profile of this emission contains the short-lived emission related to fast relaxation and trap filling that leads to the emission of the photon upon electron-hole recombination but also long-lived emission connected to charge recombination of that took longer to find an available trap state. We have shown that the latter can take much longer, especially when a significant number of excited electrons and a limited number of trap states are available.

We added to the text:

The TAS experiments highlight two pertinent aspects. First, are the real states detected within the bandgap of TiO_2 involved in the upconversion process? The ultrafast population of the conduction band with the electrons promoted via the three-photon absorption and their fast relaxation could also be rationalized using virtual states as process mediators. To test this hypothesis, similar experiments were carried out using ZnO instead of TiO_2. ZnO has a matching bandgap and is a strong emitter in the green but does not have trap states localized at 1.55 (800nm) and 3.1 (400

nm) eV from the edge of the valence band. In this case, no upconverted signal was detected, corroborating the importance of TiO_2 trap states for the process.

The second important aspect is the significant difference in photoluminescence lifetime between the sample excited at 320 nm (bandgap excitation) and via three-photon absorption at 800 nm. Excitation at 320 nm is significantly more efficient, and consequently, many electron-hole pairs are formed, leading to a higher emission quantum yield. The temporal profile of this emission contains the short-lived emission related to fast trap filling (quick relaxation) and the long-lived emission connected to charge recombination that took longer to find an available trap state. When there is a significant number of excited electrons, and a limited number of trap states are available, the trap filling can take 100s of ps even ns. This will 'delay' the emission, consistent with what was observed in figure 3. In the case of three-photon upconversion, only a few electrons are promoted compared to the number of available trap states. Consequently, only an ultrafast photoluminescence signal is present.

3) In point 4 of my initial review, I questioned the potential utility of the material the authors report for use as a window coating for building heat management. Certainly, the efficiency of this material needs to be improved if the authors ever hope to employ it to upconvert solar radiation. However, that was not my primary concern in questioning the utility of this material. Rather, I don’t fundamentally understand how transforming incident solar radiation from one color to another acts to reject any incident energy from the sun. Following upconversion, visible photons produced from near infrared light can be radiated into the building, which wouldn’t change the incident flux of energy from the sun. Moreover, converting incident near infrared light into visible light will make the windows appear to glow, which if efficient enough, could prove quite disconcerting to workers.

If the authors insist on using this idea for motivating their work, they need to do a better job in explaining why upconversion would lower the energy incident into an office building. Perhaps it needs to be coupled with some type of waveguide that could more easily reflect visible photons away from the building, or maybe direct them to solar cells embedded at the edges of the glass that could use them for power generation, etc. However, as written, the motivation for developing this material comes across as nonsensical since it doesn’t reject energy, and detracts from the results the authors report.

We thank the Reviewer for the comment. We have significantly toned down the potential application in the revised manuscript. We hope this is now consistent with Reviewer’s wishes.

jz-2023-01208g.R3

Name: Peer Review Information for "Ultrafast Infrared-to-Visible Photon Upconversion on Plasmon/TiO2 Solid Films"

Third Round of Reviewer Comments

Reviewer: 2

Comments to the Author

Overall, I think that the authors have generally done a well enough job in addressing the concerns I raised in my prior review.

Author's Response to Peer Review Comments:

Thank you very much for recognising the efforts made to improve the manuscript. We have revised the manuscript following the editorial comments.Note that no changes were made to the scientific part since the Reviewer did not request this.
